# Disseminated *Mycobacterium malmoense* and *Salmonella* Infections Associated with a Novel Variant in *NFKBIA*

**DOI:** 10.1007/s10875-017-0390-x

**Published:** 2017-04-17

**Authors:** Emily Staples, Beatriz Morillo-Gutierrez, Jessica Davies, Daniel Petersheim, Michel Massaad, Mary Slatter, Dimitra Dimou, Rainer Doffinger, Scott Hackett, Dinkantha Kumararatne, James Hadfield, Matthew D. Eldridge, Raif S. Geha, Mario Abinun, James E. D. Thaventhiran

**Affiliations:** 1 Department of Medicine, University of Cambridge School of Clinical Medicine, Addenbrooke’s Hospital, Cambridge, CB2 0QQ UK; 2 Department of Paediatric Immunology, Great North Children’s Hospital, Newcastle upon Tyne, NE1 4LP UK; 3 Cancer Research UK Cambridge Institute, Robinson Way, Cambridge, CB2 0RE UK; 4 Division of Immunology, Boston Children’s Hospital, Harvard Medical School, Boston, MA USA; 5 Department of Clinical Immunology, Addenbrooke’s Hospital, Cambridge, CB2 0QQ UK; 6 Paediatric Immunology Department, Birmingham Heartlands Hospital, Birmingham, B9 5SS UK

To the Editor:

We report a 6-year-old girl, the first child of non-consanguineous Caucasian/Thai parents, who presented at 20 months of age with disseminated, recurrent *Salmonella* enteritis with osteomyelitis and *Candida* oesophagitis. She continued to have breakthrough episodes of *Salmonella* enteritis with hematochezia and fever despite long-term parenteral antibiotics (ceftriaxone and later meropenem) and required parenteral nutrition for failure to thrive secondary to chronic diarrhea. Six months after her initial presentation, she developed a recurrent erythematous and purple skin rash with disseminated pustulonodular lesions and intermittent fever. *Mycobacterium malmoense* was isolated from the blood and skin cultures, and sapovirus and norovirus from her stools. She was commenced on combined antimycobacterial therapy (rifampicin, clarithromycin, moxifloxacin, and ethambutol) with only partial infection control (see below) and needed repeated courses of amikacin for control of exacerbations. She had been vaccinated with bacille Calmette-Guérin (BCG) at birth without sequelae. Family and perinatal history was unremarkable.

At age 3 years, she was transferred to a tertiary referral center. Examination revealed a BCG scar, skin rash (Fig. 1a), and hepatomegaly; we only subsequently noticed pointed teeth (Fig. 1b), but no other features of ectodermal dysplasia.

**Fig. 1:**
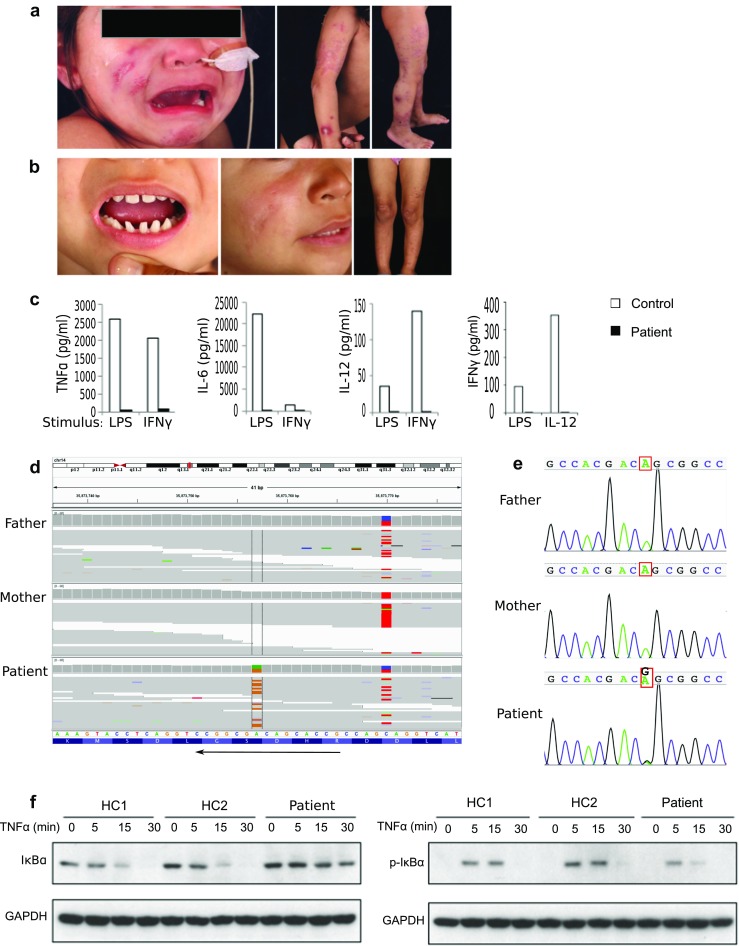
**a** Skin rash 6 months after presentation, *Mycobacterium malmoense* isolated. **b** Pointed teeth and resolution of rash at 2 years post-HSCT. **c** Concentrations of TNFα, IL-6, IL-12, and IFNγ measured in supernatants from patient and control samples following stimulation of whole blood with LPS, IFNγ, and IL-12, as indicated. **d** The genome browser view of aligned sequence data from the patient and their parents at the affected locus of the NFKBIA gene. **e** Sanger sequence traces from the patient and parents at the affected locus. **f** Western blot showing IκBα and phospho-IκBα in fibroblasts from two healthy controls and the patient following stimulation with 10 ng/ml TNFα for the indicated time points

Routine immunology blood tests (Supplementary Table 1) showed normal immunoglobulin (Ig) G and IgM levels, tetanus and pneumococcal antibodies, and lymphocyte markers with the exception that almost all (99%) of B cells were CD27-IgD+ (naive), as were the majority of T cells (no detectable CD3^+^CD4^−^CD27^−^CD45RO^+^ cells). In vitro, T cell proliferation upon phytohaemagglutinin (PHA) stimulation was within normal limits, but tumor necrosis factor (TNF)α, interleukin (IL)-6, and IL-12 responses to lipopolysaccharide (LPS) and interferon (IFN)γ stimulation of whole blood were reduced. IFNγ response to IL-12 stimulation was also reduced (Fig. 1c).

The normal response to BCG vaccination and abnormal response to IFN lead to diagnostic uncertainty regarding Mendelian susceptibility to mycobacterial disease (MSMD) with differential diagnosis including defects in either IFN or NF-kB signaling. Due to the extreme phenotype and the lack of family history, blood samples from the patient and her parents were submitted for whole genome sequencing (WGS). Illumina TruSeq PCR-free genomic libraries were prepared for each sample and aligned to the reference genome (GRCh37) using BWA v0.6.2. Genotype likelihoods were computed using SAMtools (0.1.19) and de novo mutations in the patient called with DeNovoGear (0.5.4). Variant consequences, determined using Ensembl VEP (75), were used to identify deleterious mutations. A de novo heterozygous c.94A>G missense mutation, not reported in the ExAC database [1], was found in *NFKBIA*, resulting in substitution of serine with glycine at position 32 of the IκBα protein (p.S32G) (Fig. 1d, e).

Serines at positions 32 and 36 of IκBα are phosphorylated by the IKK complex, marking the protein for degradation. The S32G variant has not previously been reported; however, S32I has been associated with immunodeficiency and ectodermal dysplasia [2, 3], and phosphorylated serine at this site is necessary for NF-κB signaling. Fibroblasts from the patient showed increased IκBα and reduced phospho-IκBα compared to controls following TNFα stimulation in vitro, confirming gain of function (Fig. 1f).

The canonical NF-κB signaling pathway regulates cellular processes including inflammation and cellular activation, proliferation, and survival. Five proteins make up the NF-κB transcription factor family in mammals: p65 (RelA), RelB, c-Rel, p105/p50 (NF-κB1), and p100/52 (NF-κB2). Each has a Rel homology domain necessary for dimerisation, DNA binding, interaction with the inhibitors of NF-κB (IκBs), and translocation to the nucleus. The prototypical p50/p65 dimer is the most abundant, present in almost all cell types, but other homodimers and heterodimers occur in specific cell types, some of which have regulatory function. IκBα is the best studied of the IκBs and constitutively binds NF-κB p65/p50 heterodimer in the cytoplasm, masking the nuclear localization sequence of p65. Conserved serines on IκBα, including S32, are phosphorylated by the IκB kinase (IKK) complex, prompting it to be ubiquitinated and degraded. The IKK complex consists of two catalytic subunits, IKKα and IKKβ, and a regulatory subunit, IKKγ, the latter also known as NF-kB essential modulator (NEMO). The other prototypical IκBs, IκBα, and IκBε also have conserved serine residues that are targeted by the IKK complex. Precursors of NF-κB1 and NF-κB2 have the carboxy-terminal ankyrin repeats that typify IκBs and can exhibit IκB activity. More recently described IκBs, Bcl3, IκBζ, IκBNS (NF-κBδ), and IκBη are found predominantly in the nucleus where they may stabilise less stable NF-κB dimers and DNA-bound dimers and may also act as transcriptional co-activators. It is perhaps not surprising given the many stimuli that can activate the NF-κB signaling pathway in many cell types with pleiotropic effects that there are multiple layers of regulation including via post-translational modifications of IKK, IκB, and NF-κB proteins [4].

Genetic defects in this pathway result in susceptibility to a wide range of infections, often with ectodermal dysplasia. Mycobacterial infections were reported in 39% of a series of 67 patients with NEMO deficiency [5] and in six of ten published cases of IKKβ deficiency [6], but only in two of ten reported cases with IκBα defects: a boy who developed a BCG abscess 7 months post-vaccination, and a girl with mycobacterial infections leading to intestinal tuberculosis, lupus vulgaris, and septic arthritis reported last year in this journal [7]. Both these patients had mutations resulting in the S36Y change in their IκBα protein. The patient we report here did not have any coding de novo variants in any other genes associated with susceptibility to mycobacterial infection.

Low production of IL-12 is consistent with the known role of the NF-κB pathway in IL-12 production by dendritic cells/monocytes and previous reports of reduced IL-12 response to the combination of IFNγ and LPS stimulation in patients with *NFKBIA* mutations [3, 8]. Janssen et al. found that the father of their patient with S32I mutation in *NFKBIA* was mosaic for the same mutation. He had a milder phenotype, but IL-12p40 response to LPS was reduced as found in his son [3]. We did not detect any evidence of mosaicism within our patient as whole blood WGS demonstrated the A to G substitution at the affected locus in exactly half the reads (11/22).

The TLR and IFNγ signalling pathways are known to interact at multiple levels. IFNγ increases TLR expression and increases expression of TLR signalling proteins including MyD88. There is also synergy at the transcriptional level, e.g., increased STAT1 phosphorylation with LPS treatment in addition to IFNγ [9]. The IL-12, TNFα, and IL-6 responses to IFNγ alone were greater than expected in the control suggesting that a low level of LPS might also have been present due to subclinical infection or contamination. In vitro studies at the single-cell level indicate that differentiation of IL-12-producing dendritic cells is characterized by activation of both the NF-κB and signal transducer and activator of transcription (STAT)1 pathways, with translocation of both transcription factors to the nucleus [10]. Reduced IL-12 responses to BCG with or without the addition of IFNγ were noted in patients with mutations in NEMO, patients with partial STAT1 deficiency, and more recently a patient with an IκBα defect [7, 11]. Patients with defects in the NF-κB and STAT1 pathways may present with similar clinical and laboratory findings.

Reported outcomes of HSCT for IκBα deficiency are not good (three died post-transplant; one was well 7 years post-transplant) [2, 7]. Following diagnosis, our patient underwent hematopoietic stem cell transplant (HSCT) at 4 years of age (bone marrow HSC graft with 3.6 × 10^6^/kg CD34^+^cells from a 9/10 HLA-A-mismatched unrelated donor, following conditioning regimen with treosulfan, fludarabine, and alemtuzumab). Throughout the transplant procedure, she continued the antimycobacterial regimen. Following initial uneventful engraftment, over the next 2 years, she developed numerous complications including (1) immune reconstitution syndrome (likely mycobacterium-related) with multiple subcutaneous nodules, fever, and raised inflammatory markers, with simultaneous EBV reactivation and significant viraemia but with no features of EBV-lymphoproliferative disease, (2) immune dysregulation with biopsy-proven “minimal change” nephrotic syndrome, strongly positive antinuclear (ANA) and chromatin antibodies, and polyarthropathy (“systemic lupus erythematosus (SLE)-like” syndrome) necessitating further immunomodulatory treatment with corticosteroids and anti-B cell monoclonal antibody (rituximab), (3) chimerism dropped from initial 100% donor to mixed, with only 1% myeloid lineage (CD15) and 40% T cells (CD3) being of donor origin. Post-HSCT, she has no palpable peripheral lymph nodes, and although her total peripheral lymphocyte count is now within the normal range, she remains very unwell. The preclinical findings demonstrating that organogenesis of secondary lymphoid structures is defective in a S32I knock-in mouse model and not correctable by transfer of wild-type bone marrow, suggests that a defect in non-hematopoietic cells may contribute to the poor HSCT outcomes [12]. The patient is now suffering from severe, treatment-resistant autoimmunity and has limited further treatment options. The potential for a 2nd HSCT will be evaluated by careful consideration of the inherent difficulty in the treatment of this condition and the continued clinical course of the patient.

This report of a novel genetic variant leading to a S32G mutation in IκBα highlights causes of diagnostic uncertainty in cases of MSMD: neither the absence of disseminated BCG following vaccination nor the elicited cytokine responses helped distinguish defects in IFN versus NF-kB signalling. It demonstrates how WGS of the family “trio” can resolve this uncertainty, by revealing genetic defects in the absence of family history and can result in rapid (3 weeks from sampling) diagnosis to inform clinical decision-making. It further adds to the experience of HSCT in this rare condition.

## Electronic supplementary material

Supplementary Table 1 (DOCX 16 kb)

## Abbreviations

BCG: Bacille Calmette-Guérin
HSCT: Hematopoietic stem cell transplant
IFN: Interferon
IKK: IκB kinase
IL: Interleukin
Ig: Immunoglobulin
LPS: Lipopolysaccharide
MSMD: Mendelian susceptibility to mycobacterial disease
NF-kB: Nuclear factor-kappa B
NEMO: NF-kB essential modulator
PHA: Phytohaemagglutinin
TNF: Tumor necrosis factor
STAT: Signal transducer and activator of transcription
WGS: Whole genome sequencing
