# Supplementary material for: Disseminated Mycobacterium malmoense and Salmonella Infections Associated with a Novel Variant in NFKBIA
Source: J Clin Immunol. 2017 Apr 17;37(5):415–8. doi: 10.1007/s10875-017-0390-x (PMC5489571; doi:10.1007/s10875-017-0390-x)
Supplement: Supplementary file 1 — (DOCX 16 kb) [file 10875_2017_390_MOESM1_ESM.docx]

**Supplementary Table 1**

| Test | Patient | Normal values (age matched)  (healthy control, for PHA) |
| --- | --- | --- |
| Haemoglobin | 85 | 115-155 |
| MCV | 62.0 | 77.0-87.0 |
| Platelets | 510 | 150-450 |
| Leukocytes | 16.53 | 5.50-15.50 |
| Neutrophil count | 4.68 | 1.50-8.50 |
| Lymphocyte count | 9.86 | 2.00-8.00 |
| Monocyte count | 1.41 | 0.70-1.50 |
|  |  |  |
| CD3+ T cells (10^9^/L) | 3.884 | 0.9-4.5 |
| CD8+ T cells (10^9^/L) | 1.357 | 0.3-1.6 |
| CD4-/CD27+/CD45RA+ (naïve) (10^9^/L) | 1.204 |  |
| CD4-/CD27-/CD45RO+ (effector)(10^9^/L) | 0 |  |
| CD4+ T cells (10^9^/L) | 2.438 | 0.5-2.4 |
| CD4+/ CD27+/CD45RA+ (naïve) (10^9^/L) | 1.592 |  |
| PHA (cpm; background => post-stimulation) | 465 => 194849 | 247 => 212048 |
| NK cells (10^9^/L) | 0.143 | 0.1-1.0 |
| B cells (10^9^/L) | 2.529 | 0.2-2.1 |
| CD27-IgD+ (naive) | 99% |  |
| CD27+IgD+ (memory) | <1% |  |
| CD27+igD- (class-switched) | 0 |  |
|  |  |  |
| IgG (g/L) | 10.4 | 4.9-16.1 |
| IgA (g/L) | <0.04 | 0.3-2.0 |
| IgM (g/L) | 1.11 | 0.5-2.0 |
|  |  |  |
| Pneumococcal antibody (mg/L) | 28 | 20-200 |
| Tetanus antibody (IU/ml) | 0.88 | 0.1-10 |
| Haemophilus B antibody (ug/ml) | <0.1 |  |
